# Transitioning of the Northwest Learning Disability (NWLD) Academic Regional Teaching Forum (HEE-NW) to a Virtual Programme With Enhancement of Medical Education for Trainees, Trainers and MDT Professionals During the COVID-19 Pandemic- a Quality Improvement Project - QIP Project Lead & Organiser -NWLD - Dr Syeda Hasan- Northwest HEE

**DOI:** 10.1192/bjo.2022.300

**Published:** 2022-06-20

**Authors:** Syeda Hasan

**Affiliations:** Pennine Care NHS Foundation Trust, Manchester, United Kingdom

## Abstract

**Aims:**

Despite the challenges posed by the pandemic, several resourceful initiatives were implemented, across the educational landscape. As the lead organiser of the academic forum the expectation was to develop a format for on-line monthly academic teachings with streamlining further innovative ideas of change to best effect. The key objective of this QIP,is to use virtual platform to establish an educational forum that is efficient, safe, reliable, easy to use and replicable

**Methods:**

- A zoom based online forum was convened using QI framework to identify gaps in the programme along with new ideas of change.
- The attendees experience new innovative practices within the programme format, in line with the original NWLD forum programme.
- A ‘think-group’ consisting of the previous NWLD forum lead, ID training programme director and higher trainee facilitators proposed ideas towards a varied educational topic bank, in line with the ID curriculum
- Qualitative feedback from the forum members collected at baseline and regular intervals.
- As part of the implementation of these actions, conducted workshops evaluating the impact of COVID-19 on Educational, Psychological and Clinical landscape

**Results:**

- There were 18 virtual sessions,5 CPD hours per session, conducted monthly.
- 9 sessions in the first PDSA cycle from June 2020 - Feb 2021, and 9 sessions in the second PDSA from March 2021-November 2021.
- Impact of COVID-19 workshops & Complex case management workshops conducted in the first and second PDSA cycle as part of the monthly academic programme.
- Participants for the academic regional teaching included Consultant Psychiatrist, higher trainees, Core trainees, Foundation doctor, specialty doctors, Medical Students and MDT professionals.
- New members regionally, nationally and internationally became part of the academic programme both as attendees and as speakers along with the Chair of the ID Faculty.
- The academic programme helped with educational and training needs. It helped in improving social participation for our members during COVID-19 pandemic.
- Qualitative feedback has been collated and analysed. The feedback contains a wealth of comprehensive information that has been used to enhance the programme and training needs for the trainees and trainers.

**Conclusion:**

- The program is well-received and led to knowledge, skill, and attitude improvements.
- The depth analysis of the feedback has facilitated a targeted approach that has brought a meaningful improvement, in the quality of training and education.
- Audience participation and engagement remains a key area for improvement.

